# Information flow among stocks, bonds, and convertible bonds

**DOI:** 10.1371/journal.pone.0282964

**Published:** 2023-03-23

**Authors:** Kihwan Jo, Gahyun Choi, Jongwook Jeong, Kwangwon Ahn

**Affiliations:** 1 Yonsei University, Seoul, South Korea; 2 SK Square, Seoul, South Korea; Massey University - Albany Campus: Massey University - Auckland Campus, NEW ZEALAND

## Abstract

This study examines the information flow between convertible bonds (CBs) and other investment assets, such as stocks and bonds. In particular, we employ transfer entropy (TE) as a proxy for the causal effect between the two assets considering that one of the most widely used methods, Granger causality, requires strict assumptions. When adopting TE, we find that asymmetric information flow arising between assets depends on macroeconomic phases. The stock and bond markets affected the CB market prior to and during the global financial crisis, respectively. In the post-crisis period, we find no meaningful information exchange between CBs and other investment assets concerning their return series. However, we observe a significant cause–effect relationship between CBs and stocks in the rise–fall patterns of their price series. The findings suggest that the appearance of one-directional information flow depends on macroeconomic conditions and the level of data, for example, return series or price fluctuations. Accordingly, investors could exploit this pattern predictability in their portfolio management. In addition, policymakers must closely monitor the information flow among the three markets. When any two markets exchange information in a state of strong market integration, unbalanced regulation between them could lead to market distortions and regulatory arbitrage.

## 1. Introduction

Rapidly growing railway companies first issued convertible bonds (CBs) in the United States during the 19^th^ century in the form of mortgage securities with conversion terms to attract investors. Companies in growing industries could finance their growth by issuing CBs that were less costly than traditional corporate bonds. Moreover, CBs offer investors the opportunity to protection against downside risks, such as those offered provided by bonds, while enjoying an upside return similar to that of a stock. When the private placement market was first introduced in the 1990s, CBs became a member of a global asset class providing investors with opportunities to select tailored securities. From 1994 to 2018, CBs exhibited performance between stocks and bonds in terms of annual average return and volatility. CBs have also exhibited a lower downside risk than stocks during stock market downturns and an upside return when interest rates have risen, and bond prices have fallen. These patterns suggest that CBs can be used to better manage risk than stock and bond portfolios and thus can be an attractive investment vehicle depending on market conditions.

This study analyzes nonlinear causality by examining transfer entropy (TE) and effective transfer entropy (ETE) between CBs and other investment assets, i.e., stocks and bonds, to determine the predictability of return series, or at least the price fluctuation patterns among these assets. Our results show how an asset could be used as a predictor for the other depending on different macroeconomic phases using information flow. Before the financial crisis, significant information flows were observed from the stock market to the CB market, and corresponding information flows were not observed from the bond market to the CB market. This finding implies that stocks can be used as a predictor to forecast CB performance. By contrast, significant information flows were observed only from the bond market to the CB market during the financial crisis, implying that bonds can be used as a predictor for CB evolution. We fail to find any causal link between the return distributions of CBs and stocks or bonds during the post-crisis period. However, the results indicate that fluctuations in the CB market can be used to predict those in the stock market. Therefore, investors could have been able to predict stock market fluctuations by monitoring CB market fluctuations, at least for the period after the crisis ended.

The remainder of this paper is structured as follows. Section 2 reviews the related literature; Section 3 describes the data and explains the methodology. Section 4 discusses the results, and Section 5 concludes the study.

## 2. Literature review

CBs have been extensively studied from various angles. However, the most common are pricing models with their issuance terms, including term to maturity [1–3], conversion provisions [4], redemption clause at maturity [5, 6], seniority of debt [7], investor puttability and issuer callability [8], interest rate [9, 10], and the relationship between CB and underlying stock [11]. In addition, several recent studies tested valuation models with historical firm data in the real world [12, 13]. Accordingly, CB valuation has become a common exercise using structural and reduced-form models that use different input variables. These studies collaborated in determining the adequacy of CB valuation. However, they are still insufficient for investors to use in constructing investment strategies, particularly in terms of sensitivity to different macroeconomic phases.

Other streams of research have been conducted from the portfolio management perspective, including CB effects on portfolio returns. For instance, [14] compared the performance of CB funds and low-grade corporate bonds. [15] adjusted the proportion of stocks and bonds in CB portfolios by decomposing the value of CBs into bond and option values. Furthermore, [16] inferred investment details through the performance of CB funds. Previous studies intensively reviewed investment strategies, such as hedging and arbitrage strategies, for improving portfolio performance [17, 18]. In particular, researchers studied asset allocation extensively by (1) employing regression models and correlation coefficients of CBs with stocks and bonds, which indicates that partial replacement of stocks with CBs could create a portfolio with an optimized risk-adjusted rate of return [19]; (2) characterizing the CB funds managed in the United States and Europe, which demonstrates that CB funds in the United States have been strongly correlated with stock returns, whereas those in Europe have been strongly correlated with bond yields [20]. However, with the increasing pace of information exchange and growing interdependency of financial markets, understanding and predicting markets to effectively manage portfolios by solely relying on correlation coefficients, regression analysis, and even the Granger causality test have become increasingly difficult [21–29]. Given that when any two markets have strong market integration with intensive information exchange, regulation risk arising from the unbalanced policy could negatively affect the market and cause regulatory arbitrage, as shown in previous studies regarding the CB market [30, 31].

In particular, Granger causality has been widely employed to identify the cause–effect relationships between financial assets, but in a very limited sense. Although the Granger causality test is commonly used to identify couplings between two systems, it is not free from criticism owing to its structural restrictions. Moreover, its use is always accompanied by a caution that its results should be carefully considered and interpreted [32]. In particular, (i) “both variables must be stationary” and (ii) “the residuals of the vector autoregression model should be Gaussian white noise” to ensure the reliability of the Granger causality test [21, 28, 29]. Unlike the Granger causality test, TE is a measure that estimates directly from data, does not require the input of any structural insights, and does not suffer from any noisy measurements of coupled dynamic systems, for example, ETE [32, 33]. Therefore, TE and ETE could be the right choice for analyzing nonlinear dependencies between dynamically coupled systems [32]: information flows between the European and US stock markets with overlapping trading hours [25]; causal linkages among virtual currencies, gold, and the dollar [28]; information flow among metals market [34]; ETE between the VIX and stock markets [26]; predicting US stock price movement using ETE with machine learning [35]; clarifying relationship among epu, investor sentiment, and stock market using ETE [36]; the information exchange between stock markets in emerging economies and foreign exchange rates [27]; group TE between cryptocurrency market [37]; information flow between new and old forks after hard forks in cryptocurrency markets [29]; the relationship among flight-to-quality, flight-from-quality occurrences, and stock-bond nexus during the pandemic era [38]; and information flow between investor sentiment and stock market [36].

## 3. Data and methodology

### 3.1 Data

The data were obtained from Bloomberg. The TR Global Convertible Index, MSCI World Stock Index, and BB Global Aggregate Bond Index were used as proxies for the CB, stock, and bond markets, respectively. Following [39, 40], we set the global financial crisis as the period from July 2007 to September 2009. A total of 1,627 trading days, covering 6 years and 3 months, comprise the global financial crisis period and 2 years before and after the crisis. Then, the data were converted into log returns because the price series has a unit root. Table 1 provides descriptive statistics for the entire sample, pre-crisis, crisis, and post-crisis periods. The whole sample period spans from July 2005 to September 2011. The three subperiods are defined as the pre-crisis (July 2005–June 2007), crisis (July 2007–September 2009), and post-crisis (October 2009–September 2011) periods.

**Table 1:** Descriptive statistics.

Panel A: Whole sample period							
Index	Obs.	Mean	Std.	Skew.	Excess Kurt.	Min.	Max.
CB	1,627	0.024%	0.508%	−0.308	4.405	−2.839%	3.756%
Stock	1,627	0.005%	1.247%	−0.285	7.963	−7.063%	9.523%
Bond	1,627	0.023%	0.364%	0.292	4.468	−1.864%	2.857%
Panel B: Pre-crisis							
Index	Obs.	Mean	Std.	Skew.	Excess Kurt.	Min.	Max.
CB	520	0.055%	0.372%	−0.142	1.419	−1.411%	1.628%
Stock	520	0.066%	0.589%	−0.266	1.322	−2.484%	2.093%
Bond	520	0.010%	0.308%	0.297	0.669	−0.964%	1.217%
Panel C: Crisis							
Index	Obs.	Mean	Std.	Skew.	Excess Kurt.	Min.	Max.
CB	587	0.006%	0.591%	−0.092	5.009	−2.839%	3.756%
Stock	587	−0.047%	1.702%	−0.109	4.823	−7.063%	9.523%
Bond	587	0.035%	0.438%	0.534	5.186	−1.864%	2.857%
Panel D: Post-crisis							
Index	Obs.	Mean	Std.	Skew.	Excess Kurt.	Min.	Max.
CB	522	0.012%	0.523%	−0.557	1.777	−2.416%	1.776%
Stock	522	0.002%	1.116%	−0.437	2.570	−5.120%	4.902%
Bond	522	0.019%	0.324%	−0.123	0.629	−1.070%	1.093%

Note. Data source: Bloomberg (https://www.bloomberg.com).

**Fig 1:**
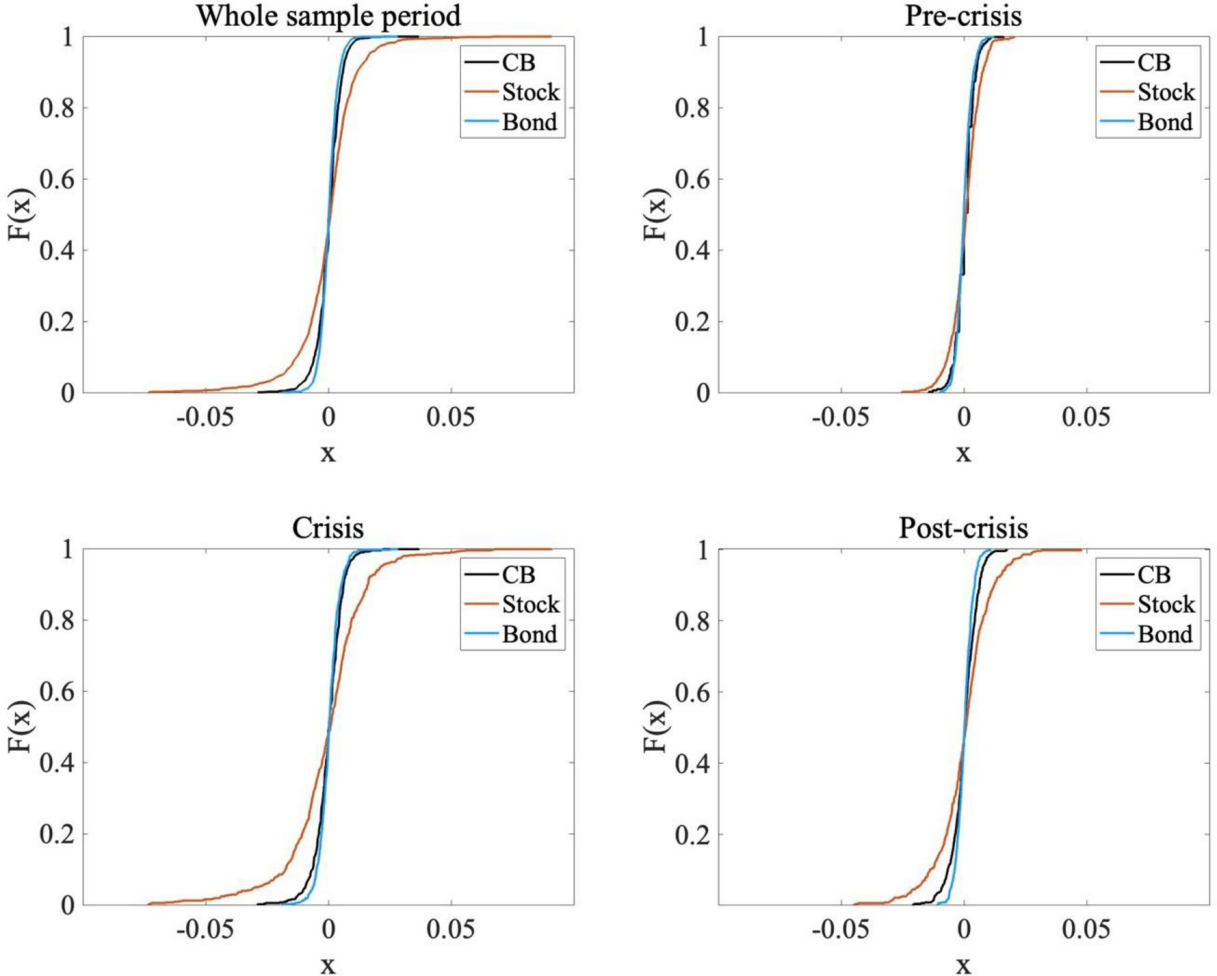
Cumulative density function of the return of CB, stock, and bond in four subsample periods: Whole sample, pre-crisis, crisis, and post-crisis periods.

Regardless of the sample period, stocks have the most volatile return distribution, followed in order by CBs and bonds. During the pre-crisis period, stocks had the highest average daily returns, again followed in order by CBs and bonds. However, bonds have the highest average daily returns during and after the global financial crisis, followed in order by CBs and stocks. For all subperiods, CBs exhibit average return and volatility between those of stocks and bonds. Stocks have a fat tail over the whole sample period [41–43], as do CBs and bonds to a lesser extent. As shown in Fig 1, the three asset classes had high excess kurtosis during the crisis period because of investor herding behavior and consequent volatility clustering [44].

### 3.2 Transfer entropy

[23] proposed the concept of TE to measure asymmetric interactions between two systems—an alternative nonlinear causality measure. TE is in an asymmetric form, which makes it possible to measure cause–effect relationships. However, the drawbacks of TE originated from computational complexity [45, 46], resulting in identification issues [47]. *X*_*i*_ and *Y*_*i*_ are considered two discrete random variables. The length of each, that is, *k* and *l*, defines $X_{i}^{\left(k\right)}=\left(X_{i},\;X_{i-1},\;\cdots ,\;X_{i-k+1}\right)$ and $Y_{i}^{\left(l\right)}=\left(Y_{i},\;Y_{i-1},\;\cdots ,\;Y_{i-l+1}\right)$, respectively. Then, TE can be defined as follows [23]:

$$TE_{Y\to X}\left(k,l\right)=H(X_{i+1}|X_{i}^{\left(k\right)})-H(X_{i+1}|X_{i}^{\left(k\right)},\;Y_{i}^{\left(l\right)}),$$

where $H(X_{i+1}|X_{i}^{\left(k\right)})$ stands for the degree of uncertainty in predicting *X*_*i*+1_ for a given $X_{i}^{\left(k\right)}$, and $H(X_{i+1}|X_{i}^{\left(k\right)},Y_{i}^{\left(l\right)})$ is the degree of uncertainty considering $X_{i}^{\left(k\right)}$ and $Y_{i}^{\left(l\right)}$ in predicting *X*_*i*+1_ Thus, *TE*_*Y→X*_ indicates the effect of $Y_{i}^{\left(l\right)}$ on predicting *X*_*i*+1_.

TE is likely to be biased because of finite sample effects, and thus, we employ ETE as a remedy. When estimating the ETE, the average TE, calculated over a finite number of independently shuffled time series, is subtracted from the observed TE. This process disrupts the temporal order in the time series and would therefore remove any possible causal relationship arising from the bias of a finite sample size. The ETE is calculated following [29, 48–50]:

$$ETE_{Y\to X}=\;TE_{Y\to X}-\frac{1}{M}\sum_{i=1}^{M}TE_{Y_{\left(i\right)}\to X},$$

where *Y*_*(i)*_ is an independently shuffled series of *Y* in the *i*-th trial; thus, ETE is demeaned TE, which is calculated by subtracting the arithmetic average of the randomized TE values from the estimated TE. Specifically, we shuffled 1,000 times and produced 1,000 TE values from independently shuffled time series over the same sample space.

We calculate the TE through histogram analysis, the most common method for dealing with discrete random variables. We use the mean squared error criterion to estimate the number of bins—an equally spaced interval of the sample range [51–53]. In addition, we use symbolic time series analysis (STSA) as an alternative method for estimating the TE. Owing to its robustness to noise, STSA is widely used in various fields, including physics, information theory, and finance [54, 55]. We use the log return data to convert each value to 0 or 1, reflecting the rise–fall pattern of the price series. Given the consecutive binary sequence, we convert the data into decimal numbers with a specific window size [56].

We then derive the significance level for TE by bootstrapping the underlying Markov process 300 times [26, 57]. The number of bootstrap replications for each direction of the estimated TE allows us to assess the statistical significance of TE estimates. Here we rely on a Markov block bootstrap proposed by [26]. In contrast to shuffling, the Markov block bootstrap preserves the dependencies within each time series. This process generates the distribution of TE estimates under the null hypothesis that no information transfer exists between two time series. Finally, we standardize the TE to a Z-score. As the mean of the Z-score is approximately zero according to the law of large numbers, the Z-score spread approximately indicates the statistical significance of TE [36, 49, 50]. In sum, we can loosely say that (i) a |Z-score| > 2.58 is in the top 1% of the critical region and hence is comparable to a *p*-value of 0.01; and (ii) a |Z-score| > 1.96 is in the top 5% of the critical region.

## 4. Results and discussion

### 4.1 Results

**Fig 2:**
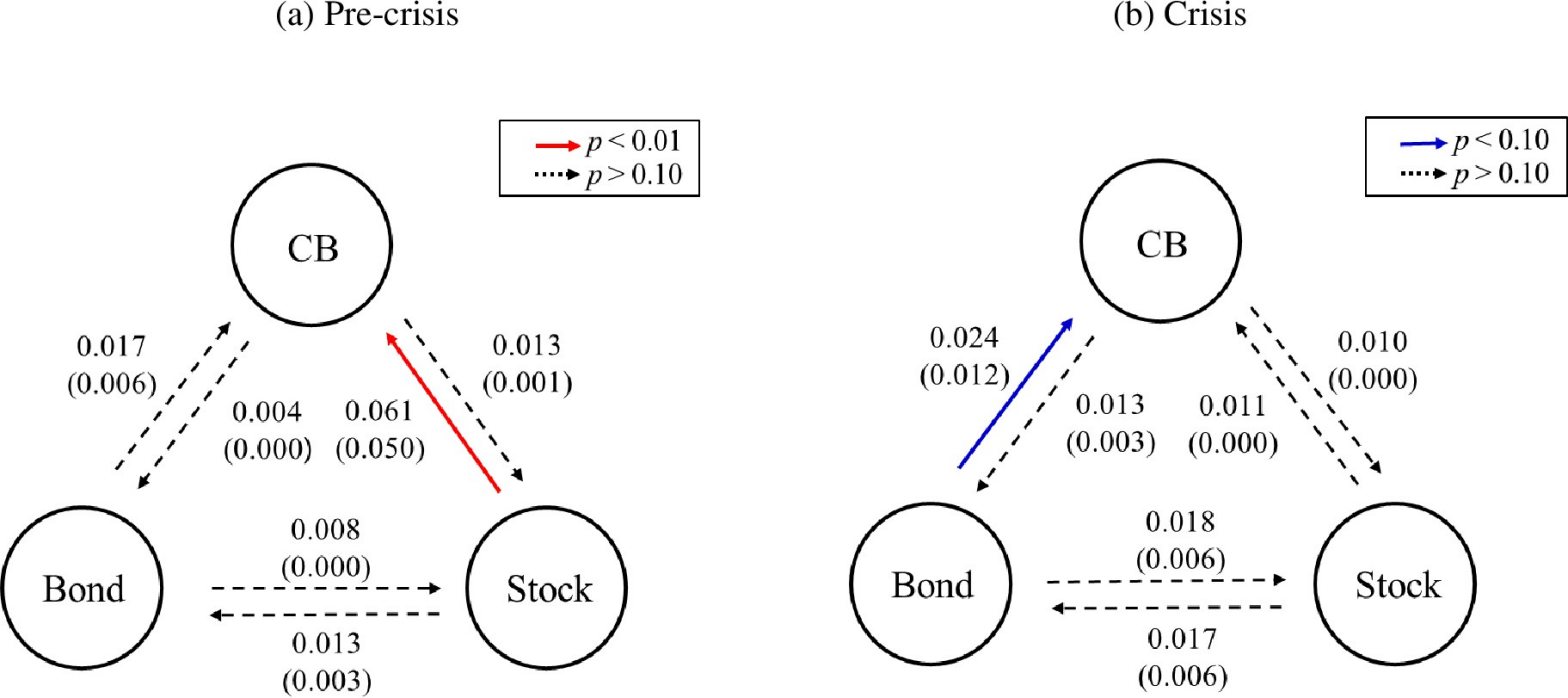
Information flow between CBs and other investment assets before and during the global financial crisis. The arrow indicates the direction of the causal link, the color represents the significance level (red and blue lines represent statistically significant information flows with *p*-value lower than 0.01 and 0.10, respectively, and the dashed line indicates insignificant TE with a *p*-value higher than 0.10), and *p* means p-value. The number denotes the estimated value of TE, and the value in parentheses stands for ETE. The significance level was evaluated by bootstrapping the underlying Markov process [26, 57]. We further apply the quantile-based TE for robustness check. In particular, cutoff points of 0.05 and 0.95 are used for the estimation (see S1 Appendix).

Fig 2(A) shows a summary of the information flow between CBs and the other investment asset classes—stocks and bonds—during the pre-crisis period. Our result indicates that statistically significant information transfer exists from the stock market to the CB market. ETE, the value in parentheses, further confirms that our results are not produced by random noise. This cause–effect relationship can be explained by the descriptive statistics reported in Panel B of Table 1. In particular, skewness and excess kurtosis—CBs are closely related to stocks. Prior studies documented that CBs illuminate the characteristics of stocks during times of continuous market rally [19] and depict the relationship between CB and the stock market [58, 59]. Therefore, we conclude that the CB market reflects the core features of the stock market along with the continued growth in the stock market since the early 2000s, *during times of continuous market rally*.

**Fig 3:**
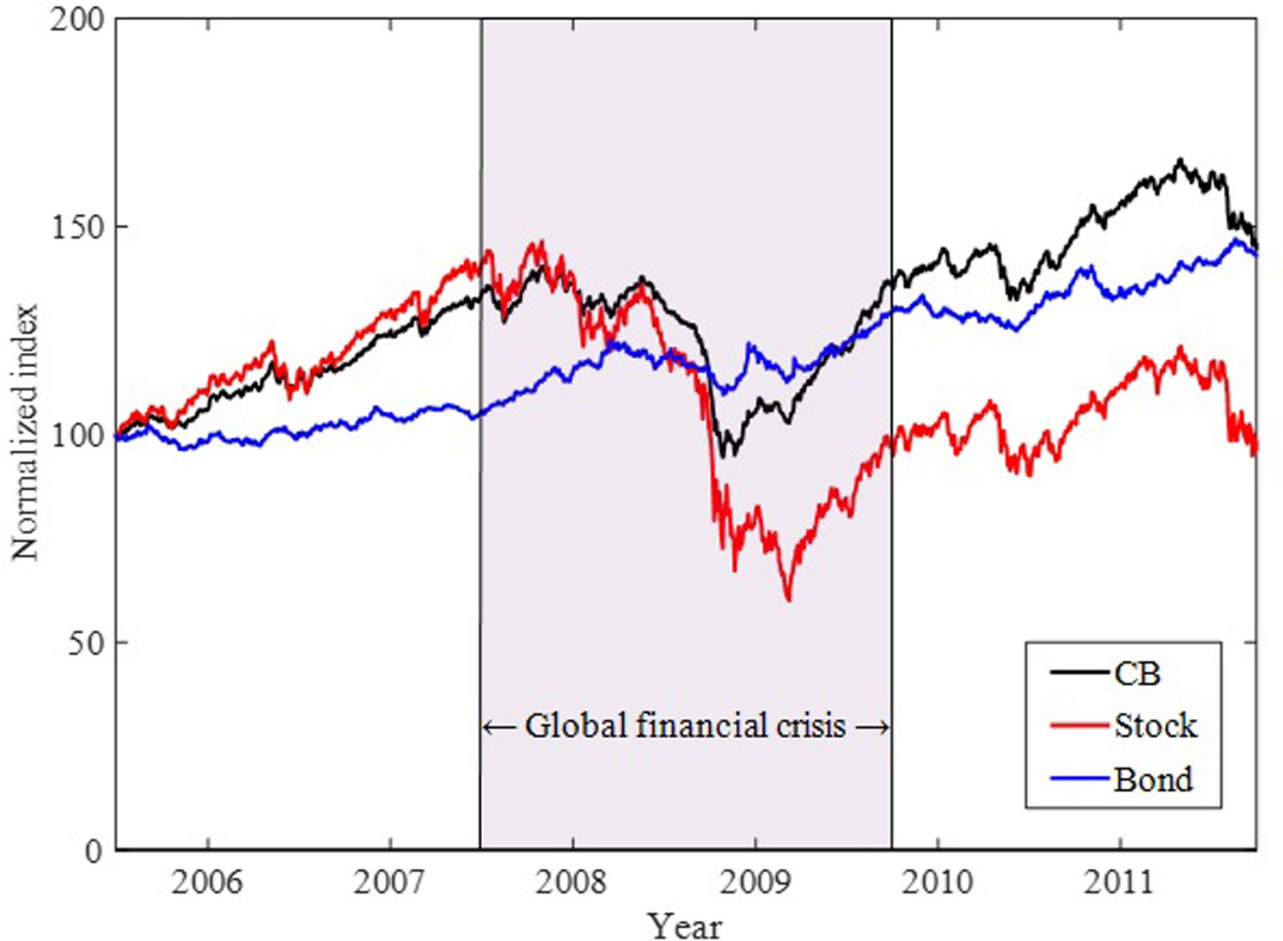
Index performance for the CB, stock, and bond markets during the sample period. Each index is normalized at 100 starting from June 30, 2005.

As shown in Fig 2(B), significant information flow occurs only from bonds to CBs during the global financial crisis, and our result is free from bias caused by finite sample effects. This finding is in line with [60], indicating that CBs showed strong bond-like characteristics in the US market during the global financial crisis. Moreover, this result is further confirmed by the conversion ratio, which was 0.543 on average during the global financial crisis [60], implying that the value of the conversion option had nearly disappeared—that is, it was out of the money. In Fig 3, the index performance of the three asset classes indicates that when the stock market struggles and falls sharply, the CB market exhibits downward rigidity and even rises with the bond market. This movement of the CB market is aligned with [61]. Thus, information flow from bonds to CBs during the financial crisis is well supported.

**Fig 4:**
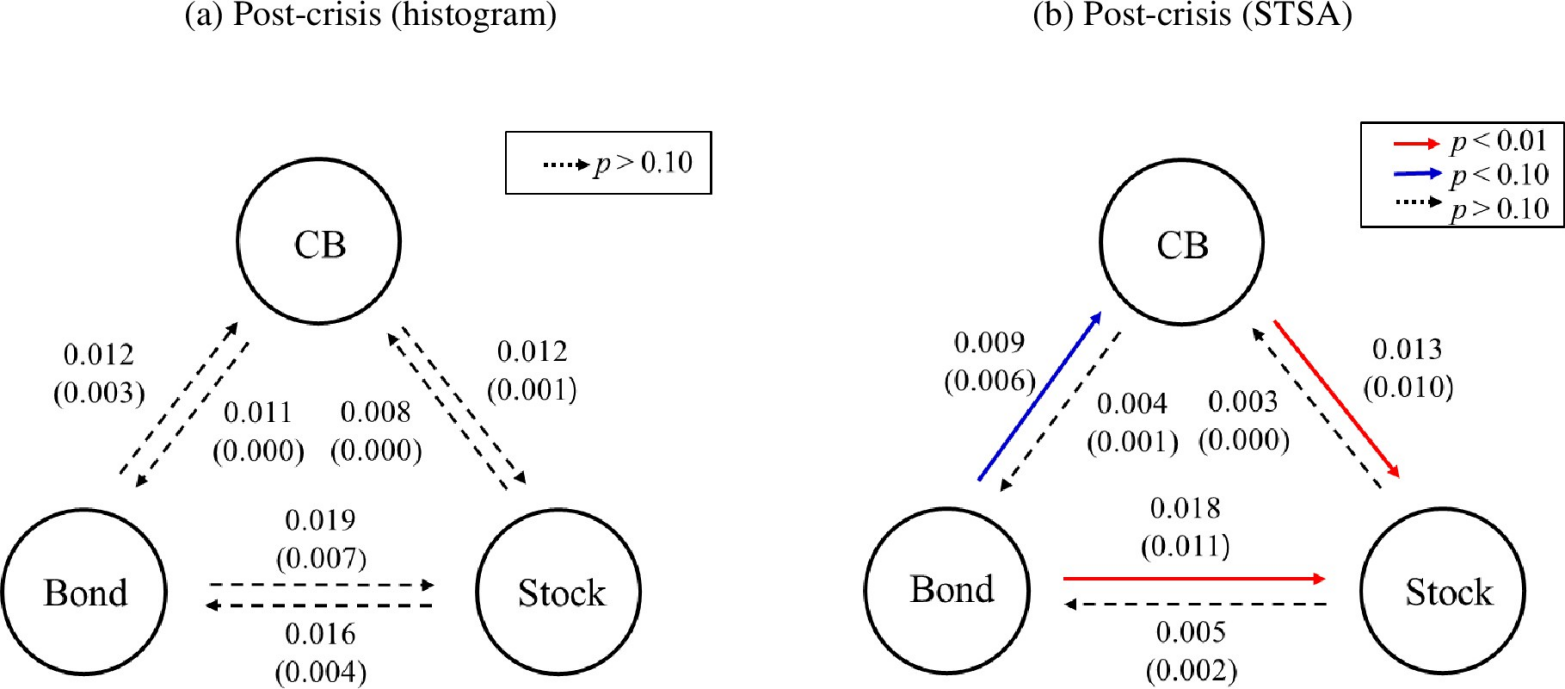
Information flow among the CB, stock, and bond markets during the post-crisis period. The arrow indicates the direction of the causal link, the color represents the significance level (red and blue lines represent statistically significant information flows with a *p*-value lower than 0.01 and 0.10, respectively, and the dashed line indicates insignificant TE with a *p*-value higher than 0.10), and *p* means *p*-value. The number denotes the estimated value of TE, and the value in parentheses stands for ETE. The significance level was evaluated by bootstrapping the underlying Markov process [26, 57]. We further apply the quantile-based TE for robustness check. In particular, cutoff points of 0.05 and 0.95 are used for the estimation (see S1 Appendix).

As shown in Fig 4(A), we cannot find any significant information exchange between the CB market and the two other markets during the post-crisis period—at least determined by employing histogram analysis between any two return distributions. Accordingly, we further investigate information flow at the level of rise–fall patterns among the different price series—that is, through STSA—and summarize the results, as shown in Fig 4(B). Specifically, TE presents two unidirectional information flows: the CB market receives information from the bond market, processes the details in its own manner, and then transmits information to the stock market. We then investigate ETE to overcome sample bias and conclude that our findings are quite robust. This result implies that the CB market is not only a transmitter of information but also a producer—the information outflow to the stock market is greater than the information inflow from the bond market.

### 4.2 Discussion

#### 4.2.1 Near-zero interest rates and quantitative easing

Information flows from bonds to other investment assets, at least about post-crisis price fluctuation patterns, are the results of interest rate cuts implemented by financial authorities as a risk-mitigation measure in times of financial turmoil [62]. A policy of monetary easing increased bond prices—bonds paying a fixed coupon become more attractive as interest rates fall, thereby driving up demand and bond prices. Near-zero interest rates and quantitative easing have stimulated economic activity, resulting in booming asset prices, for example, stocks and CBs [63–65]. In particular, the information flow from bonds to CBs suggests that an increase in CB prices reflects the bond floor well, i.e., the sum of the present value of coupon and par resulting from an increase in bond price [66].

#### 4.2.2 Lifting of regulations on short selling

Moreover, lifting regulations on short selling facilitated information flow from the CB market to the stock market during the post-crisis period. Restrictions on short selling were imposed in various countries during the global financial crisis [67]. Accordingly, arbitrage opportunities disappeared between the CB and stock markets—that is, through a combination of purchasing CBs and short-selling stocks. However, bans on short selling were lifted along with economic recovery. Thus, arbitrageurs once again provided liquidity [68, 69] to the CB market and helped reduce volatility in the stock market. Our findings are generally in line with those of prior studies, which documented that derivative markets (i) increase liquidity in spot markets [70–72] and (ii) exhibit price leadership over the corresponding spot market [73].

#### 4.2.3 Return predictions on growth stocks

TE provides details about the correlation between two time series. In particular, TE measures information transport that explicitly incorporates the directional and dynamic structure of information flow [23]. Thus, this step is essential en route to assessing causal relations between systems [74]. Accordingly, during the post-crisis period, the significant information flow—that is, at the level of rise–fall patterns in price series—from CBs to stocks implies that the price fluctuation patterns of CBs could be used to forecast the price fluctuation patterns of stocks, particularly those for growth stocks. Firms that issue CBs mostly share small and growing features, and CB funds comprise 60% small-cap growth stocks and only 40% AAA-rated corporate bonds [16]. Therefore, CB characteristics are by nature more like those of growth stocks than those of value stocks. When historically low interest rates weakened the value premium, the price fluctuation patterns of CBs led to price fluctuations in stocks. Moreover, changes in the stock market, particularly those for growth stocks, can be predicted by monitoring changes in the performance of CBs.

## 5. Conclusions

This study investigates the information transfer between CBs and other investment assets, particularly during different macroeconomic phases. We find that the stock market affects the CB market before the global financial crisis, whereas the bond market influences the CB market during the crisis. However, none of the significant information exchange between the CB market and the two other markets is found during the post-crisis period. The post-crisis period is a turmoil period with unstable and unconventional price movement. Moreover, our findings are well supported by prior studies: among cryptocurrency markets [75] and between stock and CDS markets [76]. However, we observe a significant cause–effect relationship from CBs to stocks at the level of rise–fall patterns in their price series.

The implications of our findings for investors and policymakers are as follows. First, similar predictability may be observed in the future if one-directional information flow arises depending on (i) different macroeconomic phases and (ii) different levels of data, such as return series and price fluctuations. Investors could take advantage of this pattern predictability in their portfolio management. Moreover, policymakers (regulators) must closely monitor information flow among the three markets, namely, CBs, bonds, and stocks, depending on the macroeconomic phase. When any two markets exchange information in a state of strong market integration, the unsuitable regulation between them leads to market distortions and regulatory arbitrage [30, 31, 77].

Future studies can investigate information flows between CBs and other financialized assets, such as commodities and REITs, to examine the predictability in their return series and the association of rise–fall patterns in their price series. Moreover, we can extend the data range, including more turmoil periods, and investigate the detail of the information network structure regarding different macroeconomic phases, such as the European debt crisis and cash crunch in China.

## Supporting information

S1 Appendix (DOCX)

S1 Data (XLSX)
