# Supplementary material for: Information flow among stocks, bonds, and convertible bonds
Source: PLoS One. 2023 Mar 23;18(3):e0282964. doi: 10.1371/journal.pone.0282964 (PMC10035865; doi:10.1371/journal.pone.0282964)
Supplement: S1 Appendix — (DOCX) [file pone.0282964.s001.docx]

**Appendix**

**Table 2.** Quantile-based TE result.

Panel A: Whole sample period

|  | Quantiles (5, 95) |
| --- | --- |
| CB → Stock | 0.006 |
| Stock → CB | 0.012** |
| CB → Bond | 0.007 |
| Bond → CB | 0.009* |
| Bond → Stock | 0.003 |
| Stock → Bond | 0.010* |

Panel B: Pre-crisis

|  | Quantiles (5, 95) |
| --- | --- |
| CB → Stock | 0.013 |
| Stock → CB | 0.061** |
| CB → Bond | 0.004 |
| Bond → CB | 0.017 |
| Bond → Stock | 0.008 |
| Stock → Bond | 0.013 |

Panel C: Crisis

|  | Quantiles (5, 95) |
| --- | --- |
| CB → Stock | 0.010 |
| Stock → CB | 0.011 |
| CB → Bond | 0.013 |
| Bond → CB | 0.024** |
| Bond → Stock | 0.018 |
| Stock → Bond | 0.017 |

Panel D: Post-crisis

|  | Quantiles (5, 95) |
| --- | --- |
| CB → Stock | 0.012 |
| Stock → CB | 0.008 |
| CB → Bond | 0.011 |
| Bond → CB | 0.012 |
| Bond → Stock | 0.082 |
| Stock → Bond | 0.016 |

Note. Cutoff points of 0.05 and 0.95 are used for the estimation. * and ** indicate insignificant TE with a *p*-value lower than 0.05 and 0.01, respectively.
